# From the Visible to the Invisible: On the Phenomenal Gradient of Appearance

**DOI:** 10.3390/brainsci16010114

**Published:** 2026-01-21

**Authors:** Baingio Pinna, Daniele Porcheddu, Jurģis Šķilters

**Affiliations:** 1 Department of Biomedical Science, University of Sassari, 07100 Sassari, Italy; 2 Department of Economics and Business, University of Sassari, 07100 Sassari, Italy; daniele@uniss.it; 3 Laboratory for Perceptual and Cognitive Systems, Faculty of Science and Technology, University of Latvia, LV-1586 Riga, Latvia; jurgis.skilters@lu.lv

**Keywords:** perceptual organization, Gestalt psychology, invisible visible, visible invisible, phenomenal gradient

## Abstract

**Background**: By exploring the principles of Gestalt psychology, the neural mechanisms of perception, and computational models, scientists aim to unravel the complex processes that enable us to perceive a coherent and organized world. This multidisciplinary approach continues to advance our understanding of how the brain constructs a perceptual world from sensory inputs. **Objectives and Methods**: This study investigates the nature of visual perception through an experimental paradigm and method based on a comparative analysis of human and artificial intelligence (AI) responses to a series of modified square images. We introduce the concept of a “phenomenal gradient” in human visual perception, where different attributes of an object are organized syntactically and hierarchically in terms of their perceptual salience. **Results**: Our findings reveal that human visual processing involves complex mechanisms including shape prioritization, causal inference, amodal completion, and the perception of visible invisibles. In contrast, AI responses, while geometrically precise, lack these sophisticated interpretative capabilities. These differences highlight the richness of human visual cognition and the current limitations of model-generated descriptions in capturing causal, completion-based, and context-dependent inferences. The present work introduces the notion of a ‘phenomenal gradient’ as a descriptive framework and provides an initial comparative analysis that motivates testable hypotheses for future behavioral and computational studies, rather than direct claims about improving AI systems. **Conclusions**: By bridging phenomenology, information theory, and cognitive science, this research challenges existing paradigms and suggests a more integrated approach to studying visual consciousness.

## 1. Binding Descriptions and Perceptions

Kurt Koffka [1], a prominent Gestalt psychologist, posed fundamental questions aimed at understanding how we perceive and experience the world around us. One of the central inquiries attributed to Koffka is related to the nature of perceptual experience and is summarized by the question: “Why do things look as they do?”. This question subsumes the core concern of Gestalt psychology, which focuses on the way we organize and interpret sensory information to form gestalts and meaningful perceptions [2,3,4,5,6,7,8]. It is central to understanding the principles of Gestalt psychology and reflects a deep investigation into the dynamics and mechanisms of human perception.

Koffka’s question is an inquiry into the perceptual processes and principles that determine the appearance of objects in our visual field. More specifically, it probes into perceptual organization, namely how the visual system organizes elements into coherent wholes; invariant features, i.e., how visual attributes are related to the stability of perceptual experiences under varying contexts; contextual influences, related to the way surrounding stimuli affect the perception of an object; and perceptual constancy, which explains why objects appear constant in size, shape, and color despite changes in distance, angle, and lighting.

More generally, Koffka’s question is a profound inquiry into the nature of visual perception by driving research across multiple domains, including cognitive psychology, neuroscience, and computational modeling. By exploring the principles of Gestalt psychology, the neural mechanisms of perception, and computational models, scientists aim to unravel the complex processes that enable us to perceive a coherent and organized world. This multidisciplinary approach continues to advance our understanding of how the brain constructs perceptual world from sensory inputs.

Koffka’s question is deeply related to the phenomenological approach of Gestalt psychology, which emphasizes the importance of direct, subjective experience in perception and explores how individuals perceive organized wholes directly, rather than through individual sensory inputs. This direct experience is crucial to understand why things appear the way they do. Moreover, the phenomenological approach investigates the intrinsic qualities of perceptual objects, such as form, color, and spatial arrangement, and how these qualities are seen as organized wholes. This approach helps to explain why certain patterns and structures are perceived consistently, addressing Koffka’s inquiry into the nature of perceptual experience. It also identifies and describes the laws of perceptual organization (e.g., proximity, similarity, continuity, closure, Prägnanz; [6,7,8]) ruling the way we structure our sensory inputs into coherent, meaningful wholes. Objects are not perceived in isolation but within a broader context that impart them meaning.

Methodologically, phenomenological descriptions are used to study perceptual experience without preconceived notions. These methods help researchers to directly observe and describe how perceptual phenomena appear to individuals, providing insights into why things look the way they do. At the same time, they employ carefully designed experiments to isolate and manipulate perceptual variables, observing their effects on perceptual organization [3,9,10]. This empirical approach allows researchers to validate and refine the principles of Gestalt psychology, addressing Koffka’s question through scientific investigation.

The basic role of the phenomenological approach is immediately evident when we ask, “What do you see?”. The response is typically immediate, and the descriptions are assumed to be isomorphic with the perceptual outcomes. Individuals regularly describe their perceptions without questioning the reality of those perceptions. These descriptions appear to be consistent, accurate, truthful, and exhaustive. The words used are regarded as precise labels for objects and attributes in the real world [1,2,9,11,12].

Everyday life serves as a testing ground for evaluating how well linguistic descriptions capture the essence of perceptual experiences. When discussing visual objects, people generally converge on the terminology used to describe them. It is uncommon for the accuracy of these descriptions to be challenged, except in contexts where the stakes are high and there are conflicting interests. Generally, linguistic descriptions are considered truthful and reliable, barring exceptional circumstances.

The aforementioned assertion prompts several crucial questions for our objectives. What do we describe when we see an object? What do we perceive when we see an object? Moreover, do our descriptions correspond to our perceptions, to everything we are perceiving and to everything we can perceive? If our descriptions correspond to our perceptions, to which specific perceptions and to which attributes? What specific attributes are encapsulated within these descriptions? What is visible, more visible, and less visible in what we describe and observe? More theoretically, are there inherent limitations to Koffka’s question and the phenomenological approach?

Given these questions and the previous foundational epistemological premises, the following sections will study and reevaluate the semantic content of linguistic descriptions in relation to perceptual phenomena. This analysis will extend beyond immediately perceived and described stimuli, focusing primarily on elements that elude initial perception, in order to explore the implicit, invisible, or omitted components in observers’ phenomenological outcomes.

The purpose of this approach is not to equate verbal output with the raw processes occurring in the visual system, but rather to highlight the range of perceptual attributes available to consciousness and susceptible to linguistic articulation. By mapping what participants choose to describe first, and how their descriptions evolve with prompting, we gain insight into the attributes that are more readily accessible or salient to their conscious perception, without presupposing that these verbalizations fully capture the underlying neural or cognitive operations. Titchener’s concept of the “stimulus error” serves as a historical caution, reminding us that subjective perceptual reports can conflate raw sensory input with higher-level interpretive constructs and learned categories.

The central hypothesis of this investigation posits that imperceptible and invisible elements can provide more profound insights into the structure of visual cognition than immediately perceived stimuli. Essentially, this study aims to demonstrate that a visual object, based only on the traditional phenomenological perspective, is analogous to the tip of an iceberg, with its most significant mass below the water’s surface.

In the following sections, we extend our scientific study beyond readily observable phenomena (the above-water portion), concentrating instead on the concealed, unobserved aspects. The scientific implications of this approach will be initially deduced and subsequently generalized within the framework of vision science. The main interest is to demonstrate and explore what we call “phenomenal gradient” and its implications for Vision Science.

We use the term *phenomenal gradient* to denote an empirically trackable ordering of *reportable contents* elicited by a controlled task. The ordering spans a continuum from sensory attributes (e.g., chromatic or metric properties), through perceptual organization (e.g., figure–ground, grouping), to immediate perceptual inferences that remain closely coupled to perception (e.g., amodal completion, occlusion, illumination/material cues), and finally to higher-level conceptual/event interpretations (e.g., agency or causality). Importantly, the gradient is not intended as a mere relabeling of a generic hierarchy of mechanisms; it is a descriptive construct that connects (i) the *type* of content appearing in experience/report, (ii) its *relative accessibility/salience* under minimal-report conditions (as indexed by regularities in spontaneous descriptions and their ordering), and (iii) stimulus/task manipulations that systematically shift which contents become explicit.

Reflective associations that can be generated after the initial impression (e.g., mathematical elaborations such as relating area to perimeter) are treated here as *post-perceptual conceptualization*. They belong to the far conceptual end of the continuum and are not used as evidence about early perceptual organization.

This research paradigm conceptualizes visual perception as a multi-layered process, where implicit, subliminal, or pre-attentive elements play a crucial role in shaping our conscious visual experience. By examining these hidden aspects, we aim to elucidate the underlying structure of visual cognition and perceptual organization that may not be immediately apparent in previous studies.

In other words, the phenomenological approach presented here should be regarded as an essential conceptual and empirical foundation that directly informs subsequent quantitative investigations. By systematically exploring the full range of possible percepts, including those initially invisible or merely latent, our phenomenological methods not only clarify what attributes merit measurement but also elucidate the hierarchical structure, contextual dependencies, and emergent complexities intrinsic to visual perception. This initial, structured phenomenological exploration operates as a critical precursor to psychophysical investigations, enabling researchers to identify key visual attributes, define meaningful experimental variables, and generate refined hypotheses that can then be tested using rigorous psychophysical procedures. Ultimately, phenomenology and psychophysics are mutually reinforcing: phenomenology reveals and organizes the phenomenal gradient of perceptibility, guiding the selection of attributes and stimulus conditions for quantitative assessments, while psychophysics operationalizes these insights into measurable parameters. Such an integrative strategy ensures a more comprehensive understanding of vision, bridging the gap between qualitative exploration and quantitative precision, and thereby advancing both theoretical frameworks and experimental paradigms in vision science.

Recent AI research in computer vision increasingly relies on large foundation models, diffusion-based priors, and vision–language fusion to improve robustness in challenging conditions and to inject semantic guidance into visual processing. For example, Water Cycle Diffusion leverages visual–textual fusion and diffusion-model priors for underwater image enhancement, illustrating how contemporary systems can be shaped by learned generative regularities and prompt-like conditioning [13]. While such approaches primarily target engineering goals (e.g., restoration/enhancement) rather than human perceptual organization, they provide a useful context for interpreting model-generated descriptions in our comparative framework.

## 2. Materials and Methods

### 2.1. Participants

Unless otherwise noted, each experiment described in the following sections involved different groups of 15 undergraduate students for 33 different stimuli from fields such as linguistics, literature, human sciences, architecture, and design of the University of Sassari. Participants were recruited as a convenience sample from a single university context, which may limit the generalizability of the findings. To partially mitigate narrow academic sampling, recruitment spanned multiple undergraduate study areas (e.g., linguistics, literature, human sciences, architecture, and design). Participants were naïve with respect to Gestalt psychology and unaware of the study’s specific objectives. The groups included an equal number of male and female undergraduates, all of whom had normal or corrected-to-normal vision.

All the stimuli were presented not only to human subjects but also to ChatGPT-4o, following the procedure that will be described in the next sections.

### 2.2. Stimuli

The 33 stimuli, illustrated in the following sections, were presented on a 33 cm color CRT monitor (Sony GDM-F520(Sony Corporation, Tokyo, Japan), 1600 × 1200 pixels, refresh rate 100 Hz), controlled by a MacBook computer. The overall sizes of the figures were ∼5°. The luminance of the white background was 122.3 cd/m^2^. Black and gray shapes had a luminance value of 2.6 cd/m^2^ and 51.3 cd/m^2^.

Through the stimuli used, we believe we can achieve limiting conditions that are sufficiently rich and useful for our purposes, aimed at exploring in depth the phenomenological hierarchy of vision.

Testing was conducted under controlled viewing conditions. The room illumination was provided by an Osram Daylight fluorescent light (250 lux, 5600 K). Stimuli were displayed on a frontoparallel plane at 50 cm; head position was stabilized with a chin rest and viewing was binocular. Luminance values for background and elements are reported to specify stimulus contrast under these fixed conditions.

In the case of ChatGPT-4o, the stimuli were uploaded or dragged onto the prompt screen following the procedure defined on the Web. ChatGPT-4o is a generative model that produces textual outputs conditioned on the input image and the accompanying instruction. Its responses are constrained by learned statistical regularities from training and fine-tuning (including instruction-following objectives) and can therefore vary with prompt wording and conversational context. Accordingly, throughout the manuscript we treat the AI outputs as *model-generated descriptions* rather than as direct evidence of perception in the human sense.

### 2.3. Procedure and Statistical Evidence of Perceptual Convergence

The overall procedure was divided into two complementary parts, in accordance with the classical methods traditionally employed by Gestalt psychologists (see also [1,2,3,4,5,9,10,11,14,15]). The first part consisted of a phenomenological free-report method, wherein naïve participants were asked to describe any perceptions or impressions they experienced while viewing a series of visual stimuli. The second part adopted a more quantitative approach, with participants instructed to assign percentage ratings to the descriptions obtained during the phenomenological task. This allowed for a systematic evaluation and quantification of the perceptual phenomena reported.

A phenomenological free-report method was mainly used by asking “what do you see?”. The same task was assigned to ChatGPT 4o. Separate groups, each consisting of 15 observers, described a single stimulus to prevent cross-stimulus interaction and contamination. The descriptions included in the paper were reflective of the spontaneous reports given by at least 13 out of 15 participants in each group, with edits made for brevity and representativeness. To ensure unbiased representation and avoid authorial interpretation, three independent graduate students in linguistics, who were unaware of the hypotheses, evaluated the descriptions [16,17,18,19,20,21]. These descriptions were integrated into the text to support the progression of arguments. All reports were spontaneous, and the presentation concluded when participants finished their descriptions. Participants viewed the stimuli during their reports, and observation time was unrestricted (see also [1,3,9,10,11,15]). Observation time was intentionally unrestricted in the free-report phase to allow phenomenological exploration of alternative organizations; presentation ended when participants completed their description. This choice prioritizes breadth of reported percepts over strict time-locking, and it is complemented by the subsequent rating phase that quantifies the salience of the descriptions derived from the free reports.

During the experiment, participants were permitted to reflect while observing and to perceive the stimuli in multiple ways. They could also receive questions from the experimenter in order to observe more closely and in greater depth. All potential variations occurring during free exploration were documented by the experimenter and reported in the next sections. This is necessary to determine the optimal conditions for the emergence of the perceptions under investigation.

In this section, we present the average results obtained across all experiments that will follow for each stimulus, with minor variations that are not significant for the final outcomes.

In our study, 13 out of 15 participants (86.67%) reported experiencing the described effect. The sample proportion exhibited a standard deviation of 0.34 and a standard error of 0.088. The 95% confidence interval, estimated via normal approximation, ranged from 0.695 to 1, while the exact Clopper-Pearson interval ranged from 0.679 to 0.972. A one-sample z-test against a null hypothesis of *p* = 0.5 yielded a test statistic of 3.91 (*p* = 0.00009), indicating a statistically significant deviation from chance.

In the second phase, participants quantified the principal descriptions obtained during the free-report phase by providing percentage ratings. Specifically, the groups of 15 participants were tasked with assessing the relative strength or salience (expressed as a percentage) of the primary perceptual outcomes reported in the first phase. Participants received the following instructions:

“Please indicate the extent to which your description corresponds to your perception of the stimulus, using a scale from 0 (complete disagreement) to 100 (perfect agreement)”.

In the next sections, we report only those descriptions that received mean ratings exceeding 80 across all experiments (for further details on these procedures, see [14,18,19,22,23,24]). Importantly, the statements evaluated during this phase were derived from a thorough analysis of the spontaneous reports collected earlier, ensuring that participants were not required to rate perceptual experiences that had not been spontaneously reported in the initial phase.

The average results obtained across all experiments yield a mean score of 90.73 (SD = 5.55) and a median of 90. The observed range was 18, with an interquartile range of 7. The distribution exhibited slight skewness (0.06) and kurtosis (−1.04). The 95% confidence interval for the mean ranged from 87.63 to 93.84. A Shapiro–Wilk test for normality indicated no significant deviation from normality (W = 0.98, *p* = 0.93). A one-sample t-test against a hypothetical mean of 80 yielded a significant difference (t(14) = 7.46, *p* < 0.0001), suggesting that participants reported significantly higher phenomenal evidence than the reference value.

Further statistical analyses revealed a coefficient of variation (CV) of 0.061, indicating low relative dispersion. The mode of the scores was 82, while the 10th, 25th (Q1), 75th (Q3), and 90th percentiles were 84.4, 86.5, 96, and 98.6, respectively. The standard error of the mean (SEM) was 1.43, reinforcing the precision of the estimated mean. A Box–Cox transformation suggested that data normalization was feasible (λ = 1.76), with a post-transformation Shapiro–Wilk *p*-value of 0.92, further supporting the assumption of normality. These findings confirm that participants consistently reported high levels of phenomenal evidence with minimal variability.

The LLM comparison is presented as an illustrative baseline: it contrasts human perceptual reports with model-generated descriptions obtained under the same minimal instruction (‘what do you see?’). The model outputs reflect learned statistical regularities from training and alignment and may be biased toward specific descriptive styles (e.g., literal geometric detailing) depending on these priors and on prompt/context conditions. Therefore, the present comparison does not license claims about shared perceptual mechanisms. A more controlled AI-side assessment would require repeated runs, strict prompt control, and documentation of output stability; these steps are beyond the scope of the current study and are stated as a limitation.

The LLM comparison is intended as an illustrative qualitative baseline rather than a quantitative benchmark. The model outputs are therefore reported and interpreted descriptively, and we do not compute accuracy or consistency scores. A quantitative evaluation would require (i) predefined ground-truth targets per stimulus, (ii) repeated runs under fixed prompt conditions to estimate output variability, and (iii) pre-registered scoring rules—elements that are outside the scope of the present study.

## 3. Results

### 3.1. What Do We See and What Do We Not See When We Perceive a Square?

**Figure 1:**
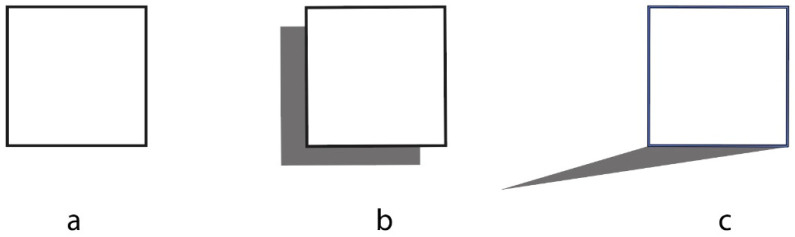
(**a**) A square; (**b**) the square is lying on a horizontal plane; (**c**) the square is standing upright arranged in the third dimension perpendicular to the horizontal plane.

Figure 1a was spontaneously described very concisely by all subjects as a square. The word “square” denotes the object being observed and describes what is currently perceived and potentially perceivable.

The description “a square” is immediate, unambiguous, and unique. No other words are needed to describe what is seen and the fact that what is seen is a square. The term “square” indicates the name of the figure and, in particular, of the perceived shape that is a square. In other words, the description refers only and exclusively to the shape. Among the many possible attributes, the shape is the one first indicated by the description. The name of the perceived object refers to its shape [25,26,27,28].

This long series of specifications might appear excessively redundant. However, it can be understood if we ask what color the square is. Starting with such a question, the same subjects, who previously stopped at just the name, showed a clear surprise, becoming aware of the coexistence of a set of perceptual possibilities. The reader might experience the same impressions [14].

The most likely answer is the following: the square is white. Immediately after, the same subjects added an alternative, albeit less immediate answer: it is transparent. A third, even less probable answer, was as follows: it is black. So, the square can be white, transparent, and black in decreasing order of immediacy. The three possible answers are placed in three layers of probable appearance. When asked to scale the visual salience of the three answers in percentage terms, the results were, respectively, as follows: 60%, 30%, and 10%.

To summarize, since the initial response included only and exclusively the shape of the object, this implies that shape is what emerges first. Color is not as immediate as shape. If the question posed to the subjects concerns the perceived color, the answer reveals a series of possibilities, with the least immediate being related to the color of the contours, which is the color of the shape. These preliminary results assume and suggest that the color of an object fills its internal region as a surface color. Despite the interior of the figure being as white as the background, within the shape the perceived color takes on qualities that have been defined as surface color [29,30]. Therefore, the interior does not appear empty but of a solid color different from that of the background (see [6]).

Phenomenally, color is not necessarily mentioned by subjects. While shape is shown in the foreground of the appearance, color lies below the foreground. From a syntactic point of view, we can state that while shape represents the noun, color appears as its adjective. It is not an accident that we nominalize the shape and mark the color adjectivally by saying “a white square and not a square white”. “White” is in all languages regarded as an attribute of the shape, not the other way around. The asymmetry between the two expressions is clearly perceptible. We suggest that this asymmetry reflects the belonging of the two attributes to two different phenomenal layers.

By definition, a noun is a part of speech that identifies a thing. Nouns are fundamental in the structure of sentences because they serve as subjects, objects, and complements. This is the case of the shape. Meanwhile, an adjective is a part of speech that describes, qualifies, or specifies a characteristic of a noun. Adjectives provide additional information and can indicate quality, quantity, size, color, origin, and other properties of the nouns they refer to. Between shape and color, the latter assumes the role of an adjective and, as such, appears in the background of the shape and can be omitted.

One could then advance the following preliminary hypothesis, yet to be confirmed, according to which the syntactic structure of spontaneous descriptions might perhaps isomorphically reflect the phenomenological structure of appearance and immediacy of perceptual qualities.

The following results demonstrate what has just been stated. If the most immediate description of Figure 1a is only and exclusively “a square”, when asking for a more detailed description, what subjects spontaneously add is “a white square”. Asking for an even more detailed description yields “a white square with black borders”. Proceeding further with the same question, the following responses are obtained: “a white square with black borders on a white background”, “a small white square with black borders on a white background”. At this point, the responses were exhausted. Despite being asked for more details, no further responses were provided.

It is worth noting that the color of the contours, and thus of the shape, in turn becomes an adjective just like the color of the square. Naturally, its position in the third plane demonstrates once again the spontaneous stratification of the phenomenology of appearance. It is fundamental to understand in what order the different perceptual qualities are arranged, which together give rise to the object in its completeness and complexity. It is crucial to ask not only how but also why the phenomenology of appearance is stratified in this way. To answer these questions, let us take further steps.

If the details provided by the subjects stop at the description of the shape and color of the square and of its contours, we must not forget a wide multiplicity of other properties visible indirectly or only a posteriori, after explicit suggestions from the experimenter. An example concerns the spatial arrangement of the square. After a clear suggestion, the mediated response from the subjects was: the square is lying on a horizontal plane. Less likely is the other possible answer: the square is standing upright, arranged in the third dimension perpendicular to the horizontal plane. In both descriptions, sketched, respectively, in Figure 1b,c, both the presence and relative position of the observer are totally implicit, who looks from above in one case, while the other observes the square in fronto-parallel vision and close to the plane on which the square stands. Yet, while we look, we not only see the objects under observation but, in all cases, also ourselves fixating on them from a precise location in space. However, being able to see oneself during observation is something relegated even further down the phenomenal gradient of appearance. The implicit and almost invisible localization of the observer can be explained by assuming seeing as a sensory modality primarily aimed at exploring the external world, its objects, and their shapes.

It is worth noting that the emergence of a new element, totally invisible explicitly but implicitly or amodally visible, is the horizontal plane. It is generated by the square regardless of the perspective in which it appears. The square does not appear to levitate or gravitate in an empty space like a planet in the universe but is supported or lying on a plane. Space becomes an object, a solid horizontal plane that hosts the square. This also occurs when the square is seen in a two-dimensional space, which is possible although less likely than the other two previously described. Under these conditions, it appears flattened on a background that is still seen as a solid flat surface containing the square.

We can therefore suggest that while in the case of color and spatial arrangement of the square, both three-dimensional and two-dimensional, we are faced with visible attributes that are nevertheless invisible or not immediately visible. In the case of the horizontal plane, it is an invisible object that becomes visible. We will return to the relationship between visible invisible and invisible visible in the next section.

It is worth noting that the element components that make up the square are visible and invisible at the same time. According to Gestalt psychologists, it is the square that emerges, that is, the shape of the whole, while the individual parts and their arrangement remain almost invisible, as if camouflaged in the background. The parts are the angles and sides of the square, but even before there are the four segments, two of which are vertical and two horizontal, they are mutually orthogonal. The component elements are also subject to stratification along a phenomenal gradient. Angles and sides are seen more in the foreground compared to the four vertical and horizontal segments. Their orthogonality is even more invisible.

The dual spatial arrangement of the square described earlier also suggests another property relegated to the lowest layers of the phenomenal gradient. This is the material of which the square appears to be composed. Even if it is not possible to specify in detail its raw material, it is instead possible to perceive that it is a homogeneous, smooth, solid and dense material, almost crystalline although not glossy. The square can also be perceived as being composed only of a sort of wire that defines its contours, while its interior is empty. In this case, the material that makes up the contours appears rigid and solid like iron.

That is not all. We have not yet covered the totality of properties distributed along the phenomenal gradient. It is indeed from the progressively less immediate properties that we can understand and scientifically appreciate the nature and the notion of what we have called the phenomenal gradient. Among all the descriptions proposed by the subjects through suggestions or when they are simply asked to say what they see, they do not contemplate, for example, the static nature of the square, which appears perfectly motionless and heavy, especially when seen lying on the horizontal plane. Other properties that are implicit and invisible at first glance are being balanced, orderly, regular, closed, harmonious, iconic, symbolic, perfect, etc.

The square can also be perceived as piece of art, as one of the most significant examples of abstract art marking a turning point in the history of modern art. The square has been a fundamental work of the Suprematist movement, founded by Malevich (see [31]), aimed at liberating art from traditional representation and move it towards a new form of expression based on simple geometric shapes and primary colors. The square, for Suprematists, represents the essence of abstraction, removing any superfluous details. For them, there is a sort of zero point of form or a tool useful to explore a modern spirituality, seeking to express the infinite and the absolute through a pure and simple form.

It also represented a provocation and a challenge to the artistic conventions of the time. These last properties of the square are not necessarily visible. For most of our subjects (60%), they are completely invisible properties impossible to be perceived even through direct suggestions.

The exploration and understanding of the phenomenal gradient can have significant scientific value. On one hand, it allows for the study of perceptual and cognitive processes and mechanisms from a new perspective. Additionally, it describes in detail the relationship between consciousness and the phenomenal gradient. Moreover, it could shed light on the evolutionary and adaptive correlates of the complex structure of perception, which can also be relevant on a comparative level between different organisms and species. Last but not least, it concerns the effects that the study of the phenomenal gradient can have in reference to computational processes related to vision and artificial intelligence.

In this regard, it is worth reporting below how an artificial subject like ChatGPT 4o “sees” and “describes” (the model output/the model-generated description) at various levels the same square previously described by human subjects. When asked “what do you see?”, the answer was “The image shows a simple black-outlined square on a white background. The square is defined by four straight lines of equal length, creating a geometric shape with 90-degree angles at each corner. The interior of the square is entirely white, with no additional features or details”.

When asking for a more and more detailed description, the answer was “The image depicts a black-outlined square centered on a white background. The square’s borders are composed of four straight, equally long lines that meet at right angles, forming four 90-degree corners. The thickness of the black outline is consistent throughout, creating a uniform and well-defined edge around the square. Inside the square, the area is completely white, without any shading, texture, or additional markings. The contrast between the black outline and the white interior emphasizes the shape and gives it a stark, minimalist appearance. The white background surrounding the square further highlights its clean and simple design, drawing the viewer’s focus directly to the geometric form”.

An even more detailed description was “The image shows a geometric figure, specifically a square, positioned centrally on a plain white background. The square is outlined with a solid black line, which is uniform in thickness, measuring approximately 1–2 pixels wide. This line forms the perimeter of the square, defining its boundaries clearly against the contrasting white background. Each side of the square appears to be of equal length, contributing to the characteristic property of a square where all sides are congruent. The corners where the sides meet are precise right angles (90 degrees), ensuring the shape maintains perfect geometric regularity. The interior of the square is filled with a pure, unblemished white, matching the background outside the square. This creates an illusion of a cut-out or an empty space defined solely by the black outline. There are no gradients, textures, or additional elements within the interior of the square, reinforcing its minimalistic aesthetic. The placement of the square on the canvas is such that it is centered, providing a sense of balance and symmetry to the overall composition. The simplicity of the design draws attention to the form itself, emphasizing the purity and clarity of the geometric shape. The overall effect of the image is one of stark simplicity and abstract minimalism, focusing entirely on the basic elements of shape and contrast. This can evoke a variety of interpretations, potentially inviting viewers to contemplate the relationship between space, form, and void”.

A first note concerns the fact that in both human and artificial subjects, a phenomenal gradient emerges, although the differences are very pronounced. What is striking is the structuring by attributes in human subjects, with shape in the foreground, then proceeding with color, spatial arrangement, and material properties up to the more expressive ones, compared to a structuring based mainly on detailing with characteristics that are more analytical geometric in the case of the artificial observer. Further comparisons through the following stimuli will allow for a deeper exploration of similarities and differences between the two phenomenal structures.

### 3.2. Variations in a Square Without Change

**Figure 2:**
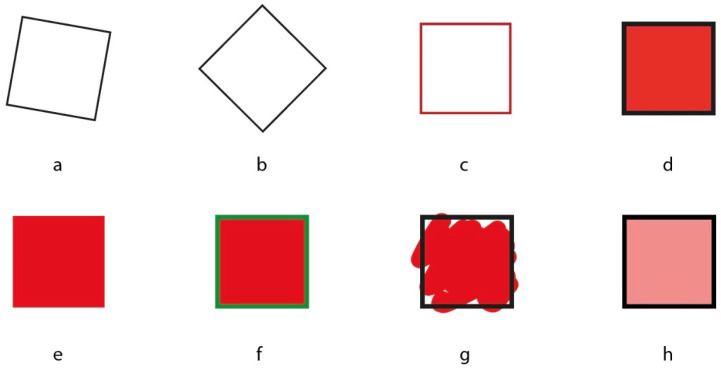
(**a**) A rotated square; (**b**) a diamond; (**c**) a red square; (**d**) a red square; (**e**) a red square; (**f**) a red square; (**g**) a red square; (**h**) a light red square.

A new group of subjects immediately describes Figure 2a as a rotated square. All the properties previously described for the square in Figure 1a have also been stratified in Figure 2a in a similar manner along the phenomenal gradient, starting from the shape up to the expressive qualities close to invisibility. What is interesting to note, in this case, is the presence of rotation, as an emerging attribute from the reference shape of the square. It is as if Figure 2a were the square from Figure 1a with a change. In other words, it is a square in the background, but what emerges in the foreground is the change: the rotation. Similarly, the artificial intelligence describes Figure 2a as follows: “I see an image of a square that appears to be tilted or rotated slightly. The square is defined by a black outline and has no other markings or colors inside”.

Figure 2b was described as a rhombus or a diamond. When asked “why is it seen as a rhombus despite being a square rotated by 45 degrees?”, the most common answer was “the square shows the sides, while the rhombus shows the edges. While the first appears flattened, the second appears pointed” [31]. In the case of the AI, the answer was “The shape appears as a diamond because it is a square that has been rotated 45 degrees. In its standard position, a square has sides parallel to the horizontal and vertical axes. When rotated, the corners of the square point up, down, left, and right, creating the appearance of a diamond. This change in orientation gives the square a different visual perception, making it look like a diamond instead of a typical square”. The AI provided a more geometrically accurate explanation focusing on the rotation and axes.

Human subjects describe Figure 2c as a square. Only after a more detailed request do they report seeing a square with red contours. When asked “what color is the square?”, the most common answer was white, then transparent, and finally red. The AI responds: “The image shows a simple red square with a thin border on a white background. The square is centered in the image”. By asking “what is the color of the square?”, the answer was unique: the color of the square is red.

Human perception is more complex and articulated, as well as more syntactically structured. This indicates that human perception follows a kind of syntax or hierarchical organization. In contrast to the AI’s straightforward description, humans organize visual information in a way that is similar to how language is structured, with primary and secondary elements. Humans initially focus on the overall shape (a square), then notice specific features (red contours) when prompted and then have varying interpretations of the square’s color (white, transparent, red).

For human subjects, Figure 2d appears very simply as a red square. Only after specific questions was the black border mentioned. Artificial intelligence describes the stimulus as follows: “the image shows a red square with a black border on a white background. The square is filled with a solid red color, and the black border is relatively thin. The square is centered in the image”.

These results highlight several interesting points. First of all is simplicity vs. detail. While humans provide a simple, essence-capturing description, the AI offers a more analytical description of the image. The AI output biases a black border, which was not mentioned in the human description. This could indicate that the boundaries are specifically related to the shape and not to the color. Human brain specializes the roles of different attributes, i.e., if one component of the object (e.g., the boundary contours) drives the shape, then it cannot show another attribute like the color and vice versa (see also [31]).

Moreover, while the AI includes information about the square’s position (centered) and the background color, the human description does not mention them. More generally, humans seem to abstract the essential information (shape and primary color) while ignoring what they might consider non-essential details (border, background, positioning).

It is worth noting that to highlight certain attributes, it is necessary to make less important attributes implicit or hidden, attributes that are less relevant for defining what is seen in the simplest and most immediate way. Some attributes are more significant in defining the identity of an object compared to others. Shape is more identity related, constant, and immutable in defining an object compared to its color, which, however, is more so than the spatial position or other characteristics present in the lower layers of the phenomenal gradient.

In other words, to show something, it is necessary to hide something else, hence hiding in order to show. To bring something to the foreground, other perceptual qualities must be pushed to the background. More generally, to see something, it is necessary to make something else invisible, i.e., placed in the background. This creates a true computational economy of seeing, where the most important things come first, then the rest. This can be a starting hypothesis to explain the formation of the phenomenal gradient.

For human subjects, Figure 2e–h are also red squares, although in the case of Figure 2f, the description for a small portion of the subjects (30%) becomes a red square with green edges. In the case of Figure 2g, it is not necessary for the color to fill the entire square shape for the square to appear red [23,31]. Figure 2h appears as a light red square. Now, the attribute “light” becomes an adjective of the color, i.e., less salient than color within the phenomenal gradient. While the red color is mentioned by all subjects, it being light is not equally immediate, being mentioned by only half of the subjects.

The related immediate descriptions of the AI are as follows. Figure 2e: “The image shows a red square on a white background. The square is completely filled with red and has no visible borders”. Figure 2f: “I see a square with a green border, and the inside of the square is filled with a solid red color. The green border is relatively thin compared to the red area inside. The red color is uniformly distributed within the boundaries of the square”. Figure 2g: “I see a square with a black frame, and inside it, there is a red blotch. The red blotch does not have a defined shape and appears to be spread randomly within the square”. Figure 2h: “A black square outline or frame. Inside the frame is a solid light red or pink square that completely fills the interior space. The edges of the inner square align perfectly with the black frame, creating a neat, geometric appearance. The color of the inner square is uniform, without any variation or texture visible”.

The comparison between humans and AI highlights again the previous points: simplicity vs. detail, role specialization of shape and color, and human abstraction of essential information vs. AI geometrical details. Another noteworthy result concerns the visible emergence of invisible objects such as the horizontal plane, previously described, which is absent in the artificial subject. Beyond structuring appearance along a phenomenal gradient, a peculiarity of human vision is the formation of invisible objects that give meaning, generate some form of consistency, justify and explain what we perceive, as well as the formation of the phenomenal gradient itself. In the following section, changes capable of eliciting emerging visible invisibles will be introduced.

### 3.3. Changes and Emerging Objects Within a Square

The stimuli, described in the previous section, have not brought about significant changes from a phenomenal point of view, apart from Figure 2a,b. This is mainly because there are no evident dissimilarities along the shape of the reference square. In fact, the chromatic variations do not generate any real change or any new emerging object. This is presumably due to the position of color in the background of the phenomenal gradient compared to emerging shape. Introducing extensive changes and dissimilarities along the shape of the square is supposed to represent the main source for the emergence of significant phenomenal changes of a totally different nature compared to color. This would further corroborate the hypothesis of the phenomenal gradient as a place or structure where objects and their attributes are organized.

Across stimuli that primarily manipulated local geometry, the model’s descriptions largely remained at the level of explicit shape/color. For stimuli designed to elicit amodal completion and event interpretations in humans, human reports frequently included completion/material/causal attributions, whereas model outputs tended to (i) restate visible geometry and (ii) provide less stable event-level interpretations.

Unlike color, dissimilarities along the shape of the square would no longer represent adjectives providing additional information but would be true emerging objects, not detached or totally segregated from the square but in close relationship with it. We will see in this section how this occurs.

**Figure 3:**
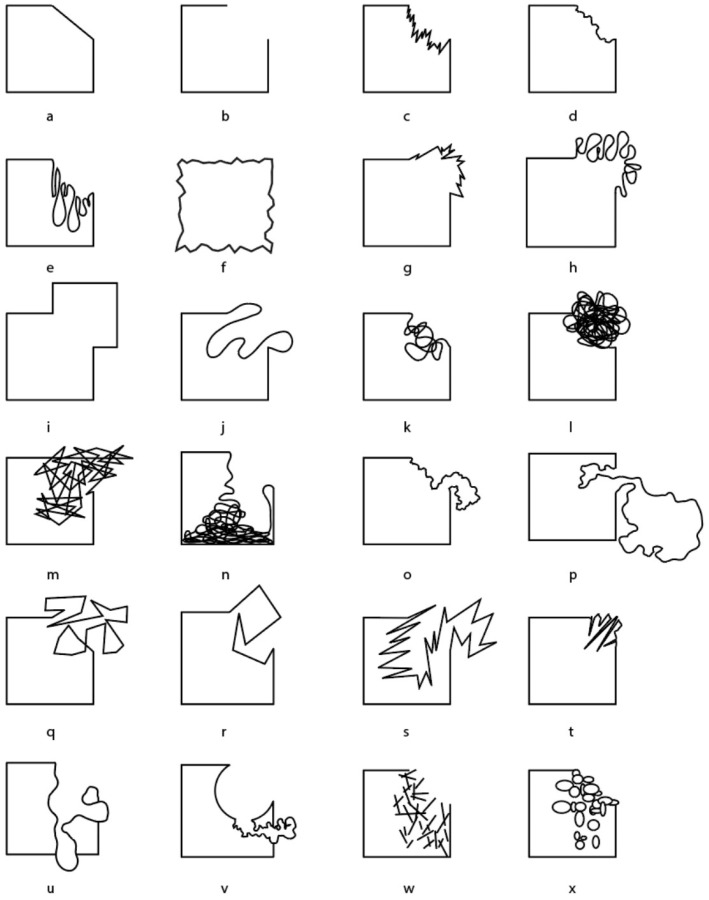
For human and AI perceptions and descriptions, see the text.

Figure 3a was spontaneously described as a rounded square and, with more details along the phenomenal gradient, as a square of white paper with a corner clipped by scissors. Qualitatively different is the AI’s description: “the image shows a geometric shape that resembles a square with one corner cut off. It has five sides, making it a pentagon. The top right corner of the shape is truncated, giving it a distinctive appearance compared to a regular pentagon or square. The lines are black, and the shape is outlined against a white background. More in details, the image is a geometric figure with the following characteristics: it is a five-sided polygon (pentagon); the bottom side is horizontal and of a certain length; the left side is vertical and equal in length to the bottom side; the right side is also vertical but slightly shorter than the left side; the top side is horizontal, matching the length of the right side; the top right corner is cut off, resulting in a slanted line connecting the top and right sides; all lines are black, and the shape is outlined clearly; the shape is set against a plain white background. The truncated corner gives it a distinctive appearance, making it different from both a regular square and a regular pentagon”.

The pentagon does not appear in human descriptions although it is visible, albeit in the background of the phenomenal gradient. Moreover, several visible invisibles emerge: the paper, the cause of the cut, and especially the scissors. Causality becomes the phenomenal explanation, the justification for the fact that an amodal square is seen even though modally it is and can be perceived as a pentagon [19]. In other words, the cause of the cut, in turn caused by the scissors, justifies and imparts meaning to the square and the change that are immediately seen.

These visible invisibles are part of how humans organize visual elements to create meanings from what they see, filling in gaps amodally with assumed new information. Amodal completion refers to the human tendency to perceive complete shapes even when parts are occluded, obscured or missing [20,32,33]. In other terms, the human mind quickly creates a causal chain (the corner was cut with scissors) to complete and explain what it sees. The emergence of causality imparts meaning to the perception and completes and justifies why we see a square with a cut corner rather than simply a pentagon.

The AI does not engage in this kind of causal visual inference. It describes what it literally sees without making assumptions about how the shape came to be or what it might represent. The AI provides a much more geometrically precise description, identifying it as a pentagon and giving exact details about side lengths and angles without the emerging phenomenal interpretation placed in the foreground by humans.

In short, humans typically report completion, material, and causal/event interpretations, whereas the model tends to generate descriptions emphasizing local geometry and explicitly visible components. We interpret this divergence as reflecting different inductive biases and constraints: human reports arise from perceptual organization and perceptual inference, while model outputs arise from learned textual/image–text regularities and instruction-following objectives.

Figure 3b is perceived as the perimeter of an incomplete square with a missing corner. AI describes the figure as follows: “The image depicts an incomplete square or rectangle. Here are the detailed characteristics: the figure is an incomplete square or rectangle with three complete sides and part of a fourth side; the bottom side is horizontal and fully drawn; the left side is vertical and fully drawn, perpendicular to the bottom side; the top side is horizontal but only partially drawn, starting from the left side and stopping before reaching the right side; the right side is vertical but only partially drawn, starting from the bottom side and stopping before reaching the top; all lines are black, straight, and of equal thickness; there is a clear gap in the top and right sides, making the shape incomplete; the bottom-left corner forms a right angle (90 degrees); the bottom-right and top-left corners also form right angles, but the lines do not extend to complete the angles on the top and right sides; the background is plain white with no additional elements or markings”.

The human description is concise and meaningful by immediately identifying the shape as a square, despite it being incomplete. This shows the human tendency to recognize and complete familiar shapes, even when parts are missing. On the contrary, the AI provides a much more detailed, geometric description by focusing on describing exactly what is visible, including the lengths of lines, their orientation, and the angles formed.

This comparison highlights further differences between human and AI output biases. Humans tend to use prior knowledge to interpret and complete shapes, while AI describes what it sees more literally and in greater detail. The human perception demonstrates concepts like Gestalt principles and amodal completion, while the AI shows a more analytical, element-by-element approach to description.

Moreover, even if both outcomes acknowledge the incompleteness of the shape, the human description implies a complete square with a missing part, while the AI describes it as an incomplete figure without perceiving and assuming what it might be if completed. The human description is more holistic, focusing on the overall concept rather than specific details. While the AI explicitly mentions the white background and the absence of additional elements. The human description does not mention this component, focusing solely on the main shape. ‘First things first’ can be considered a useful simplified motto to understand the formation of the phenomenal gradient.

Figure 3c appears as a square plate of a glass-like material with a corner shattered by a violent blow imparted by an object similar to a hammer. In the case of the AI, (for reasons of brevity, we omit the purely geometric description similar to that of the previous and subsequent figures concerning the sides and angles, the lines, the vertices, and the background) the description is as follows: the image shows predominantly a square with one side partially eroded or jagged. The jagged section has multiple peaks and troughs, creating a saw-tooth effect. The exact number of peaks and troughs varies, and they are irregular in length and angle.

This stimulus reveals a clear difference between visual interpretation and description, more particularly, it shows several visible invisibles: a causal phenomenon, a violent blow from an object similar to a hammer. On the contrary AI makes no causal inferences, but only describes the visible features. Moreover, humans perceive specific material, glass-like, that implies a 3D object by using the term plate. AI describes it as a 2D shape: a square.

These comparisons have significant implications for AI development, particularly in areas like computer vision and natural language processing. They suggest that to achieve more human-like perception and description, AI systems might need to incorporate the following: causal reasoning capabilities, knowledge of materials (see [34]) and their properties, understanding of real-world physics, ability to make contextual inferences, and integration of multi-modal information (visual, tactile, functional). All of this underscores the complexity of human visual processing and interpretation, which goes far beyond pattern recognition to include aspects of physics, causality, and real-world knowledge.

Figure 3d is described as a square of soft material as if it had been gnawed by some rodent. The AI description was as follows: the image shows a square with one side partially eroded in a wavy or uneven manner. The figure is predominantly a square, but with an irregular, wavy section that has multiple curves, creating a natural, flowing effect. Again, for brevity, the geometrical details have been removed, being similar to the previous AI descriptions and to the following.

These differences continue to suggest the gap between human perception, which readily incorporates holistic real-world knowledge and causal inference, and AI analysis, which focuses on detailed geometric description without making inferences about materials, causes, or real-world contexts.

Figure 3e shows a rubbery square that is melting and dripping in one corner due to heat. The AI description: “The image shows a square with one side partially eroded in a looping or irregular manner. Multiple loops of varying sizes and shapes creates a whimsical, flowing effect”. These strong differences continue to highlight the challenges in developing AI systems that can interpret images in ways that align with human perception and understanding. They also underscore the depth and complexity of human visual processing, which goes beyond pattern recognition to include aspects of physics, causality, and real-world knowledge.

Figure 3f shows a sheet of paper that has been crumpled by hand and then reopened. For the AI description, the image shows a square with jagged, irregular edges resembling to a square that has been roughly cut or torn.

Especially in this figure, but actually in the others as well, another visible invisible object is time. In all the illustrated cases, there is a component that appears as the background, a square, from which some kind of event or change emerges. While amodally completing the square, just as the perception of a figure amodally completes the background, the change and dissimilarity along the shape gives rise to new emerging objects and qualities that appear as visible invisibles. As a matter of fact, allowing the amodal completion of the square implies generating the notion of time, which can only be linked to that events. Time and changes, dissimilarities that can be more generally defined as “happenings” are phenomenally two realities that imply each other, two sides of the same coin. When one is present, the other is always present as well. One cannot be perceived without the other. When something happens, even under static conditions, it is presumed that there is a past, a present (crystallized in the image under observation), and a future, which can be seen as still in progress or concluded.

Talking about happening implies giving meaning to something much simpler, which we can call much more aseptically “dissimilarity”. All the events illustrated in Figure 3 are dissimilarities, mostly corresponding to the top right corner, appearing as changes and, more phenomenally as happenings. These dissimilarities are conspicuous, true objects emerging from the background of the square, unpredictable and unexpected conditions that become the true source of new information. The dissimilarities become visual novelties by activating a chain of other emerging qualities that justify and bring together similarities and dissimilarities, making the former totally homogeneous, thus generating the square, and accentuating the latter, i.e., by making them to assume a new and emerging meaning. This kind of complex organization, structured within the layers of the phenomenal gradient, generates whole information where all the components of the visual field appear meaningful and useful. In this way, the complex relation between similarity and dissimilarity within the same figure puts together all the elements, reducing uncertainty and entropy, thus adding value to a decision-making process.

Time is a visible invisible, and the concept of time, though not physically present in static images, is perceived and inferred by human observers, but it is not reported in any of the previous and following AI descriptions. In addition, the fact that the square is perceived as a background, with changes or events emerging from it, highlights the visual tendency to separate stable elements from dynamic or dissimilar elements in a scene. Since changes and dissimilarities are perceived as new, emergent information, it suggests that our perceptual system is particularly attuned to novelty and difference.

More generally, regions of dissimilarity in the image (changes, irregularities) contain more information in the information-theoretic sense. They introduce unpredictability, increasing information content. The regular, unchanging parts of the square are instead highly predictable, thus containing less information. Moreover, the visual dynamics of deriving emergent meanings from dissimilarities can be seen as the outcome of information processing. Thus, reducing uncertainty by introducing new emerging objects and cause on the dissimilarities, useful in explaining and completing the square on the background, is a way visual perception reduce entropy.

Amodal completion (perceiving the complete square) can be considered as a form of information compression that reduces the complex visual input to a simpler, more familiar concept (a square with a modification). Novel or unexpected elements (like the happenings in the square) have high information value. However, the complementation and consistent integration between the completion of the square and the emerging meaning of the happening, useful in explaining the amodal square, is the most effective way to reduce the information load and the entropy of the visual world. The reduction in entropy occurs through three different ways: reduction in the unexpected and uncertain multiplicity of the elements that compose the dissimilarity, completion of the square through the segregation of the happening, and, finally, complementation and bringing together of the square and the happening in a concise and compact form.

We suggest that human vision is a mechanism aimed at reducing the entropy of the uncertain multiplicity of fragments and dissimilarities of the perceptual world. This aligns with information theory principles [35]. On the contrary, AI descriptions seem to increase the entropy compared to a human observer. The different lengths of the human and AI descriptions corroborate what has just been stated.

These theoretical notes suggest that AI systems might benefit from being designed to be more sensitive to areas of high information content (dissimilarities, unexpected elements) in images. Moreover, the phenomenal structuring of visual information into background and figure elements could be a useful principle in developing more sophisticated image segmentation algorithms. Further theoretical notes will be discussed through the phenomenology of the following figures.

In Figure 3g, a strange eruption or small explosion manifests in the top right corner. AI description: the image shows a square with a section near the top right corner modified by a jagged, sawtooth-like pattern. The jagged extension consists of several sharp, angular points that protrude from the top right corner of the square. The extension begins near the top middle part of the square and continues outward through the top right corner, extending slightly downward along the right side. The pattern resembles a sawtooth or lightning bolt effect with sharp, defined points.

In Figure 3h, the square appears as a sort of inflorescence or a growing of living matter starting from its interior. AI description: The image shows a square with a section near the top right corner modified by a wavy, looping pattern extending outward. The looping extension consists of several interconnected loops that extend outward from the top right corner of the square. The loops are smooth and continuous, giving the appearance of an organic, flowing design. The extension begins near the top middle part of the square and continues outward through the top right corner, extending slightly downward along the right side.

Interestingly, both descriptions note the organic quality of the pattern, though they express this in different ways. These observations continue to highlight key aspects of human vs. AI perception. Humans readily interpret static images in terms of dynamic processes (growth, expansion), while AI descriptions remain more static. Human perception integrates knowledge from various domains (biology, physics) to make sense of visual patterns. AI provides more detailed geometric descriptions but does not make inferences about what the patterns might represent in the real world. These findings suggest again the following areas for potential improvement in AI systems: developing AI that can infer dynamic processes from static images; integrating multidisciplinary knowledge to interpret visual patterns; enhancing AI’s ability to recognize and describe organic qualities in a more holistic way; developing AI that can infer potential origins or causes for observed patterns.

In Figure 3i, the square seems to be multiplying or self-cloning, starting from one of its corners. The AI description: The image shows a square with an additional geometric shape attached to the top right corner. The additional smaller square is rotated and appears to be protruding out of the main square. The connection between the two squares forms additional angles and intersections, creating a complex geometric figure.

The contrast between these descriptions continues to highlight the challenges in creating AI systems that can interpret images in ways that align with human perception. It also underscores the rich, inferential nature of human visual processing, which goes beyond simple pattern recognition to include concepts of process, causality, and analogy.

In Figure 3j, the square appears to be fluttering like a sheet moved by the wind blowing mostly on one of its corners. AI description: The image shows a square with an irregular, wavy extension protruding from the top side and top right corner. The wavy extension consists of two prominent, smooth curves that protrude from the top side and the top right corner of the square. The larger curve extends from the middle of the top side towards the right, while a smaller curve protrudes from the top right corner, giving the appearance of fluid, organic shapes.

In Figure 3k, a corner of a thread-like square seems to be knotting itself, twisting on itself. AI description: The image shows a square with a small, irregular pattern of loops near the top right corner. The tangled pattern consists of several overlapping loops creating a small, chaotic appearance. The loops intersect and overlap in a seemingly random manner, forming a loose, web-like structure. The pattern is concentrated near the top right corner but does not extend far into the square.

In Figure 3l, the top corner of the square tangles up, creating a sort of chaotic circular ball of yarn. The AI description: The image shows a square with a dense, tangled pattern of loops concentrated at the top right corner. The tangled pattern consists of numerous overlapping loops creating a dense, chaotic appearance. The loops intersect and overlap in a seemingly random manner, forming a complex, web-like structure. The densest part of the pattern is located in the top right corner, with loops extending slightly towards the center and less densely towards the other parts of the square.

These descriptions suggest further key aspects of human vs. AI perception. Humans readily infer material properties and apply associated behaviors to abstract shapes. Visual perception often interprets static images as moments in dynamic processes, while AI tends to describe static configurations. Vision may attribute agency or intentionality to inanimate objects in images, while AI describes patterns as passive features. Human descriptions often focus on the overall concept or implied action, while AI provides more detailed geometric descriptions. Humans frequently use analogies to familiar objects or actions to understand and describe abstract shapes. AI can provide more objective descriptions of complexity and extent, while humans might imply these through action-based descriptions. Human vision integrates knowledge of physical behaviors and properties, while current AI systems typically rely solely on visual data.

In Figure 3m, a square appears as if crystals are rapidly growing in the top right, gradually filling it. AI description: The image shows a square with a section inside filled with a complex, intersecting line pattern, mainly concentrated at the top right corner. The intersecting pattern consists of numerous straight lines crossing each other at various angles, creating a dense, chaotic appearance. The lines intersect in a seemingly random manner, forming a complex web-like structure. The densest part of the pattern is located in the top right corner, with lines extending towards the center and less densely towards the other parts of the square.

In Figure 3n, the contours of a square seem to unroll like a thread and pile up at its base. AI description: The image shows a square with a section inside filled with a chaotic, tangled line pattern. The tangled pattern consists of numerous overlapping and intersecting curved lines, creating a dense, chaotic cluster at the bottom. Some lines extend upward from the cluster, forming a looser, more open tangled pattern. The right side has a notable loop extending out from the dense cluster, creating an additional irregularity.

In Figure 3o, a square made of foam seems to be losing matter that slowly detaches from one of its corners. AI description: The image shows a square with a small, irregular, wavy extension protruding from the top right corner. The extension appears to be a small, amorphous, wavy shape that starts from a small wavy section at the top right corner of the square and expands outward. The edges of the extension are smooth and flowing, with no sharp angles or straight lines, giving it a natural, organic appearance.

In Figure 3p, the square seems to be erupting much more matter than it appears to contain. The matter that comes out seems to slowly inflate like a sort of balloon. AI description: The image shows a square with a large, irregular, wavy shape extending from its top right corner. The extension appears to be an amorphous, blob-like shape that starts from a small wavy section at the top right corner of the square and expands outward. The edges of the extension are smooth and flowing, with no sharp angles or straight lines, giving it a natural, organic appearance.

In Figure 3q, the square seems to be faceting, breaking, or projecting large pieces outward. AI description: The image shows a square with several additional geometric shapes attached to and extending from the top right corner. The additional shapes are a mixture of polygons with varying numbers of sides and angles. These shapes overlap and intersect in some areas, creating a visually complex pattern that extends outward from the top right corner of the main square.

In Figure 3r, a piece of the square is torn off and broken by an invisible force. AI description: The image shows a square with an additional geometric shape attached to the top right corner. The additional smaller square is rotated and appears to be protruding out of the main square. The connection between the two squares forms additional angles and intersections, creating a complex geometric figure.

Human perception often infers causes for observed effects, even when the cause is not visible in the image. In addition, we may infer damages or changes to the integrity of original shapes, while AI tends to maintain the original shape and describe additions to it. Human perception may introduce invisible elements to explain visible effects—in Figure 3r, it is an invisible ‘visible invisible’ thing or force—while AI sticks strictly to describing visible elements.

In Figure 3s, a square is broken through and shattered in a zigzag pattern by something unknown, maybe an electric shock. AI description: The image shows a square with a section featuring a jagged, lightning-bolt-like pattern extending from the top right corner. The jagged section has sharp, angular lines that zigzag from the top side down to the right side, creating a dramatic, lightning-bolt-like effect. The lines vary in length and angle, creating a chaotic and dynamic appearance.

In Figure 3t, a square brick of stone, perhaps marble, with a corner damaged or shattered due to a received blow. AI description: A square with a section of one side modified by a pattern resembling cracks or jagged lines, creating a chaotic pattern.

In Figure 3u, a square with the top part melting and seeming to empty out, but it strangely appears full at the bottom. The emptying part seems to be simultaneously filling up. The flowing void appears full. It is an impossible figure. AI description: A square with a large, irregular, wavy extension protruding from the right side, disrupting the straight lines of the right and bottom edges, resembling a fluid or blob-like shape.

While humans perceive paradoxes or impossibilities (simultaneously empty and full), AI describes what is visually present without noting logical inconsistencies. Human vision explores and checks consistency within the visual world. This applies not only to human vision but also to animal vision. Consider, for example, an antelope constantly on alert, attentive to verifying the consistency that random noises do not imply the presence of a predator. For its part, the leopard must be able to assess the consistency of its own random noises compared to those produced by the surrounding environment in order to camouflage them effectively.

In Figure 3v, the square shows a missing circular surface with an appendage similar to an intestine that, while being empty, is simultaneously also full. AI description: A square with one side partially removed in a combined smooth and irregular wavy pattern, resembling a large semicircle cut out from the top side.

Figure 3w features a square that shatters into many tiny pieces similar to twigs or segments that fall to the ground. AI description: A square with a section filled with intersecting lines, resembling a crisscross or shattered pattern, creating an irregular and chaotic appearance.

In Figure 3x, the square, made of oval components similar to stones, seems to collapse in the top right corner, perhaps due to its weight or because they are not cemented. AI description: A square with one side partially filled with a cluster of ovals of various sizes, overlapping and clustering together in the top right portion. In these latest figures, for simplicity, all detailed geometric descriptions related to the different components of each stimulus have been omitted.

The comparison continues to highlight the rich, inferential nature of human visual processing, which integrates real-world knowledge, causal reasoning, and even the ability to perceive logical paradoxes.

To complete this section, let us make some general theoretical considerations. Our results might have significant implications for neuroscience, particularly in understanding the neural mechanisms underlying visual perception and cognition. The suggested phenomenal gradient, where shape is prioritized over color and other attributes, suggests a hierarchical organization in attribute visual processing. This aligns with current understanding of the visual cortex’s organization, where different visual features are processed in distinct areas and integrated at higher levels. Our study may help refine our models of how information flows through the visual system, from primary visual cortex (V1) through higher-order visual areas.

The human ability to perceive visible invisibles and complete partially occluded shapes might support the role of top-down processing in vision. This implicates higher cortical areas, including the prefrontal cortex and parietal areas, in shaping visual perception. Future neuroimaging studies could investigate how these areas interact with visual cortices during the perception of ambiguous or incomplete visual stimuli.

Moreover, the tendency to make causal inferences from static images suggests involvement of brain areas associated with causal reasoning, such as the medial prefrontal cortex and temporoparietal junction. This highlights the need for neuroscientific investigations into how these higher cognitive areas interact with visual processing regions during perception of complex scenes.

The human tendency to make causal inferences from static images might suggest involvement of brain areas associated with causal reasoning, such as the medial prefrontal cortex and temporoparietal junction. This highlights the need for neuroscientific investigations into how these higher cognitive areas interact with visual processing regions during perception of complex scenes.

Our results can also have several implications for computational theories of vision and artificial intelligence. The phenomenal gradient supports the use of hierarchical models in computer vision, where different features are processed at different levels of abstraction. This aligns with the success of deep learning models in computer vision tasks, which inherently implement a form of hierarchical processing.

The strong difference between human and AI interpretations, particularly in causal reasoning, suggests a need for integrating causal inference capabilities into computational models of vision. This might involve developing hybrid models that combine traditional computer vision techniques with causal reasoning frameworks. In addition, the ability to perceive visible invisibles and make context-dependent interpretations suggests that computational models can be designed to integrate contextual information and prior knowledge more effectively, such as by developing more complex attention mechanisms or incorporating memory systems into visual processing models.

Our findings support the idea that visual perception can be understood as a process of entropy reduction. This aligns with information theory approaches to perception and cognition, suggesting that computational models should be designed to optimize information compression and extraction of meaningful patterns from visual input.

The ability of humans to infer causes and complete partial shapes might support the Bayesian view of perception as a process of combining sensory evidence with prior knowledge [31]. The study suggests that human priors are rich and multifaceted, incorporating not just statistical regularities of the visual world, but also causal knowledge and expectations about physical processes. The observed phenomenal gradient also suggests that Bayesian models of vision should be hierarchical, with different levels corresponding to different attributes (shape, color, etc.) and their relative importance in perception. Since humans tend to interpret static images as snapshots of dynamic processes, Bayesian models might incorporate dynamic priors—expectations about how scenes and objects change over time, even when presented with static input. The ability of humans to generate coherent interpretations of ambiguous stimuli supports the Bayesian concept of explaining away, where the presence of one cause for an observed effect reduces the probability of alternative causes. This suggests that Bayesian models of vision should implement complex explaining away mechanisms to account for human-like interpretations of visual scenes.

Our outcomes can also be reconsidered in terms of evolutionary theory, providing insights into the adaptive value of human visual processing. The phenomenal gradient may reflect an evolutionary adaptation for quickly identifying objects in the environment. Rapid shape recognition would be crucial for detecting predators, prey, or other significant environmental features. The human tendency to make causal inferences and perceive happenings even in static scenes may have evolved as a way to predict and prepare for future events. This ability would be highly adaptive in a dynamic environment where anticipating changes could mean the difference between survival and peril.

The ability to perceive visible invisibles and complete partially occluded shapes could have evolved as a mechanism for dealing with partially obscured objects in natural environments. This skill would be crucial for identifying partially hidden predators or prey. The observed tendency of human vision to reduce entropy by generating coherent interpretations of ambiguous stimuli may reflect an evolutionary pressure for cognitive efficiency. By compressing complex visual information into simpler, meaningful representations, the brain can make rapid decisions with limited cognitive resources.

The rich interpretations made by human observers, often involving inferences about texture, material properties, and physical processes, suggest that human vision evolved to integrate information across multiple sensory modalities. This cross-modal integration would be highly adaptive in a complex, multi-sensory environment.

## 4. Discussion

This work explored the nature of visual perception through a comparative phenomenology of human and artificial intelligence (AI) responses to a series of modified square images. Our findings reveal significant differences in how humans and AI systems process and interpret visual information, shedding light on the complex nature of human visual cognition and the current limitations of AI in replicating human-like perception.

Because the LLM comparison is qualitative, it cannot provide a systematic metric of human–AI ‘perceptual’ distance. We treat the AI outputs as model-generated descriptions shaped by learned priors and prompt conditions, and we use them only to highlight interpretive contrasts (e.g., tendency toward literal geometric description vs. humans’ completion- and event-level interpretations). Developing a quantitative benchmark for this paradigm is left for future work.

Our results support the existence of a phenomenal gradient in human visual perception, where different attributes of an object are organized hierarchically in terms of their perceptual salience and importance. Shape consistently emerged as the primary attribute, followed by color, spatial arrangement, and other properties. This hierarchical organization, although aligned with Gestalt principles of perception [1,7], introduces a distinct form of perceptual organization and suggests that human vision prioritizes certain attributes and information to efficiently process complex visual scenes.

The mere order in which attributes are reported may not be an entirely reliable proxy for perceptual salience. Educational and cultural factors, such as exposure to certain tasks (e.g., geometric exercises), may predispose observers to prioritize shape over color. Indeed, such influences could shape the default “phenomenal gradient” and may reflect learned heuristics rather than intrinsic perceptual hierarchies. Follow-up studies that employ systematically varied instructions, stimuli of different complexity and relevance, or observer populations with differing cultural and educational backgrounds are needed.

The “phenomenal gradient”, as defined in this work, refers to a hierarchical, multi-layered organization of perceptual attributes and qualities that emerge during the act of seeing. In this conceptualization, certain attributes, such as shape or orientation, manifest immediately and with high salience, forming the perceptual “foreground”. Other attributes, such as color nuances, material properties, causal interpretations, or contextual inferences, remain initially less conspicuous or even invisible, residing in progressively deeper layers of the perceptual field. As attention, probing questions, or contextual cues are introduced, these more subtle attributes are brought into conscious awareness, effectively “surfacing” from lower levels of the phenomenal gradient.

Importantly, post-perceptual associations (e.g., mathematical relations such as area–perimeter) are treated here as conceptual elaborations that can follow perception, and thus belong to the deepest, least immediate layers of the gradient rather than to early perceptual organization.

Technically, the phenomenal gradient can be construed as an implicit, adaptive ordering of visual information. It is not a static or universal hierarchy; rather, it is dynamically modulated by factors such as observer experience, task demands, cultural exposure, and contextual relevance. This gradient thus operates as a perceptual framework for organizing visual inputs along a continuum of immediacy, clarity, and interpretive richness. From an information-processing perspective, the phenomenal gradient can be seen as reflecting underlying neural and cognitive mechanisms that prioritize certain stimulus properties for rapid encoding and interpretation, while relegating others to a latent but accessible state, ready to be “activated” through directed attention, suggestion, or extended perceptual analysis.

In essence, the phenomenal gradient provides a structured conceptual model for understanding how complex perceptual outcomes arise from layered, interacting levels of visibility, salience, and interpretative depth. It offers a tool for analyzing and comparing how different individuals, or even artificial systems, parse and integrate incoming sensory information into coherent visual experiences. Beyond the general sensation–perception–conceptualization continuum, the phenomenal gradient provides an explicit, report-based operationalization that separates organization/inference contents from later associations, enabling targeted stimulus manipulations and psychophysical tests that selectively probe each region of the continuum.

This study demonstrated a remarkable ability to perceive visible invisibles—objects or properties not physically present in the image but visually inferred based on context and prior knowledge. This phenomenon, closely related to amodal completion [2], was particularly evident in cases where subjects perceived causes for observed effects (e.g., scissors cutting a corner) or completed partially occluded shapes. These findings support theories of top-down processing in visual perception [36] and highlight the constructive nature of human vision.

Human observers consistently made causal visual inferences and interpreted static images as snapshots of dynamic processes. This tendency to perceive happenings rather than mere geometrical configurations is aligned with Gibson’s ecological approach to visual perception [37], emphasizing the importance of action and event perception in human vision.

The human visual system’s ability to generate coherent interpretations from ambiguous or incomplete visual information can be understood as a form of entropy reduction [38]. By inferring causes, completing shapes, and attributing meaning to visual patterns, the human brain effectively compresses complex visual input into simpler, more manageable representations. This process agrees with predictive coding theories of perception [39] and suggests that human vision is optimized for extracting meaningful information from noisy or ambiguous sensory input.

These insights have implications across multiple disciplines. In neuroscience, they suggest the need for further investigation into the neural mechanisms underlying hierarchical visual processing and top-down modulation. In computational theory, they highlight the importance of integrating causal reasoning and context into visual processing models. From a Bayesian perspective, they underscore the richness of human priors and the need for dynamic, hierarchical Bayesian models of vision. Evolutionarily, they point to the adaptive value of rapid shape recognition, causal inference, and entropy reduction in visual processing.

There are some points, already mentioned in previous sections, that nevertheless deserve further elaboration in this section.

The concept of visual syntactical organization represents a novel way of understanding visual perception, bridging insights from linguistics, cognitive psychology, and neuroscience. It offers a rich framework for future research into the structure and processes of visual consciousness. Implications for the science of consciousness are the following. The phenomenal gradient suggests that conscious visual experience has a hierarchical structure. This aligns with theories of consciousness that propose multi-level or hierarchical models, such as the Global Workspace Theory [40,41,42,43,44] or Integrated Information Theory [45,46,47,48,49,50,51]. It suggests that consciousness might not be a unitary phenomenon but a structured, multi-layered experience. Our work demonstrates that conscious visual experience goes beyond passive reception of sensory data. The perception of visible invisibles and causal inferences implies that consciousness actively constructs our experience of reality. This supports constructivist theories of consciousness that view it as an active, generative process [52,53,54,55].

The richness of interpretations and inferences made by participants, even from simple stimuli, relates to the debate about phenomenal overflow [56,57,58,59,60,61,62], the idea that we consciously experience more than we can report or attend to. This study suggests that visual consciousness might indeed be richer and more detailed than traditional theories assume. The ability of participants to combine sensory input with prior knowledge and make complex inferences supports theories of consciousness that emphasize information integration. Moreover, the tendency of subjects to perceive static images as snapshots of dynamic processes aligns with predictive processing theories of consciousness [39,55,63,64,65,66], which propose that conscious experience is fundamentally about predicting and making sense of sensory input. The prevalence of causal inferences in subjects’ responses suggests a deep connection between consciousness and causal reasoning, potentially supporting theories that view consciousness as fundamentally involved in modeling causal structures in the environment.

In the previous section, we mentioned the implications of our outcomes for information theory significant as applied to visual perception. Traditional information theory, developed by Claude Shannon [35], focuses on the statistical properties of signals and their transmission. However, the rich and complex nature of human visual perception revealed in this study suggests that a more multidimensional approach to visual information may be necessary. The phenomenal gradient, where shape takes precedence over color and other attributes, suggests that visual information is processed hierarchically. This implies that not all bits of visual information are equally weighted in human perception, contrasting with the uniform treatment of information in classical information theory.

The ability of humans to perceive visible invisibles and make causal inferences suggests that contextual information plays a crucial role in visual perception. This contextual information is not present in the stimulus itself but is added by the observer, challenging the traditional sender–receiver model of information theory. The tendency of humans to interpret static images as snapshots of dynamic processes suggests that temporal information is inferred even from static visual input. This implies that the information content of a visual stimulus may be greater than what is explicitly present in the physical signal. The human ability to generate coherent interpretations from ambiguous or incomplete visual information can be seen as a form of lossy compression. This aligns with rate-distortion theory in information theory (see [67,68]) but suggests that the distortion in human perception may be more complex than simple signal degradation. The generation of additional information (e.g., causal inferences, completion of occluded shapes) by the human visual system suggests a kind of ‘negative entropy’ where the perceived information exceeds the information content of the physical stimulus.

Based on these implications, we might propose a new notion of visual information that extends beyond the classical bit-based concept to encompass the rich, multidimensional nature of human visual perception. We term this new concept “Visual Information Units” or VIUs. VIUs are multidimensional units of visual information that incorporate hierarchical weighting on the position in the perceptual hierarchy (e.g., shape-related VIUs carry more weight than color-related VIUs). The information content of a VIU is modulated by contextual factors, including the observer prior knowledge and the surrounding visual context. VIUs incorporate a temporal dimension, reflecting the potential for dynamic interpretation even in static stimuli. VIUs include a causal component, reflecting the tendency of human perception to make causal inferences. VIUs account for amodal completion, where perceived information exceeds physically present information.

Formally, each of these components would be a function of both the stimulus properties and the observer prior knowledge and cognitive state. This notion of visual information has several implications. It provides a framework for quantifying the richness of human visual perception, including aspects that go beyond the physical stimulus. It might guide the development of more human-like AI vision systems by providing a framework for incorporating hierarchical processing, contextual modulation, and causal inference. It can suggest new approaches to perceptual compression in image processing, potentially leading to more efficient and perceptually accurate compression algorithms. It can make specific predictions about neural processing of visual information.

Going back to the Introduction section of this work, the results of our study reveal several limitations in the traditional Gestalt phenomenological approach and in Koffka’s question, “Why do things look as they do?”. While these frameworks have been invaluable in advancing our understanding of visual perception, our results suggest that they may not fully capture the complexity and richness of human visual experience.

Gestalt principles largely deal with visible elements and their relationships. However, our study highlights the importance of visible invisibles, elements that are not physically present in the stimulus but are nevertheless perceived. This includes inferred causes, completed shapes, and imagined processes. In addition, while Gestalt psychology acknowledges the role of past experience in perception, it does not fully account for the complex causal inferences that our subjects made. Observers inferred causes and processes that were not directly represented in the images.

Traditional Gestalt principles treat all perceptual attributes more or less equally. However, our study reveals a clear hierarchical structure in perception, the phenomenal gradient. A related Koffka-like extended question could be as follows: “How are different visual attributes hierarchically organized in our perceptual experience?”.

While Gestalt psychology provides rich descriptive accounts of perceptual phenomena, it does not fully engage with information concepts. Our study suggests that visual perception can be understood as a form of information processing, involving entropy reduction and the generation of negative entropy through inference and completion.

Modern theories of predictive processing and active inference suggest that perception involves constant prediction and error correction. The traditional Gestalt approach does not fully incorporate these dynamic, predictive aspects of perception.

Based on the findings of this study and the limitations we have identified in Koffka’s original question, we can formulate a new, more comprehensive question that might better captures the complexity of visual perception as revealed by our research. This new general question represents a significant evolution from Koffka’s original formulation. It encapsulates the key findings of our study, addressing the complex, constructive nature of visual perception that goes beyond mere organization of sensory input. The question “How does the human visual system construct meaningful, dynamic representations of the world from sensory input, prior knowledge, and inferential processes?” acknowledges perception as an active, constructive process, recognizes the importance of meaning and interpretation in perception, includes the dynamic aspect of perception, even of static stimuli, and includes the role of prior knowledge and inference. This new question could serve as an organizing principle for future research in visual perception, encouraging a more integrated approach that combines phenomenology, information theory, predictive processing models, and neuroscientific investigations.

In summary, the stark differences between human and AI perception underscore both the sophistication of human vision and the challenges that remain in developing truly human-like artificial visual systems. By continuing to explore these differences and integrate insights from cognitive science, neuroscience, and computer vision, we can work towards bridging the gap between human and machine perception. It underscores the intricate relationship between perception, cognition, and information processing, pointing towards a more integrated, multidisciplinary approach to the study of vision. Our comparison identifies recurring divergences between human reports and model-generated descriptions—particularly regarding causal/event interpretations, amodal completion, and material/agency attributions. We treat these divergences as concrete targets for future work (both empirical and computational), rather than as evidence that the present study directly advances AI system design or decisively refines existing theories of vision.

To conclude, there remains an important point to discuss. Based on what has been stated in the previous sections, one might think that the phenomenal gradient is a rigid structure, a sort of a priori or a chain of immutable assumptions or postulates of reality from which objects and their properties are constituted in a true syntactic structure. Not at all. On the contrary, the phenomenal gradient and the results obtained suggest that it can be understood as a flexible structure, where different attributes can be remodulated and redistributed in its various more or less superficial layers. For example, in certain circumstances, we can certainly and easily put color in the foreground compared to shape. Think of the color of fruits when they ripen or when we choose them at the market, a color that emerges even before the shape. Think also of the color of a dress or a particularly captivating female make-up based primarily on chromatic accentuation. Animal camouflage is another trick to highlight color against shape. This also applies to the material qualities of an object in relation to its color or shape. Moreover, expressive qualities can also be in the foreground compared to shape, as happens, for example, in art where even the most minimalist, shapeless work still becomes an expressive perceptual object above all else.

Saying this does not mean affirming that the phenomenal gradient is entirely subjective and random. It is and must be a well-formed basic structure, a prior, a necessary default reference, a structure that is nevertheless flexible, a given that precedes any other possible variation but that still allows modifications based on evolutionary, social, cultural, and even subjective needs. All this also applies to language, here understood as a reflection of the gradient, which, while having a precise syntactic structure, allows variations according to which color, usually understood as an adjective of the form that represents the noun by default, in certain circumstances can emerge in the foreground compared to the shape. This happens when, for example, we affirm “the red of the square, the red of the lips, the color of the eyes”.

Speaking of flexibility does not mean saying that any reversals lead to a chaotic restructuring. On the contrary, it is meant to suggest that such changes occur according to precise rules that concern above all and perhaps only and exclusively the qualities to be remodeled, while the others not considered remain on the same phenomenal plane or level assigned to them by default. In summary, the phenomenal gradient does not belong only to the domain of statics but is part of dynamics, and it is precisely in this dynamic that consciousness plays an even more significant and fundamental role, which can reshape the structure of the gradient by bringing out what is initially found in depth or at the bottom. The opposite can also happen when, for example, a fortuitous reversal of the gradient or just because someone has made us notice something invisible activates the previously dormant consciousness resting in the default gradient.

A clear and simple demonstration of all this is given in the present work and, more precisely, in the results relating to the square in Figure 1, where the questions posed to the subject allowed for remodeling of the phenomenal gradient, bringing out visible invisible attributes such as the material qualities and spatial arrangement of the square, as well as its expressive qualities, which the subject initially failed to perceive as qualities placed in the deepest levels of the phenomenal gradient and, therefore, far below the surface. The reader himself may have manifested a feeling of bewilderment or illumination as if something had been overturned in speaking of certain qualities that were invisible at first glance. But this is nothing other than awareness. Overturning or modifying the default phenomenal gradient implies modifying the state of consciousness, of visual awareness. In this case, perceptual reality is changed, assuming new and emerging meanings.

Perceiving art, in essence, means nothing other than intervening and reshaping the phenomenal gradient. Making a square become art, as happens in Malevich’s Square, is equivalent to what has been stated. This also happens within science, where great discoveries are often linked to the emergence of attributes or visible invisible objects previously completely invisible. The dynamics of the phenomenal gradient is probably at the basis of creativity, intelligence, learning and consciousness. If this were true, then understanding the laws and principles of its dynamics takes on scientific relevance and priority.

Therefore, there is still much work to be done in order to better understand not only the structure of the default phenomenal gradient but also the laws and principles of its dynamics that allow it to be creatively reshaped. What has been suggested and discovered in this study constitutes only the tip of an iceberg, whose main mass is completely submerged and of which we perceive only a small part. Nevertheless, it may have been important to grasp at least its existence, although largely still submerged.

A limitation of the present study is that participants were recruited from a single university and represent a relatively homogeneous demographic and cultural context. While this is common in initial experimental phenomenology and helps control task comprehension and logistics, it constrains external validity. Replication on more diverse samples (age, educational background, and cross-cultural/language contexts) is needed to test the stability of the reported ‘phenomenal gradient’ patterns and to evaluate the extent to which report ordering and content are shaped by language and cultural learning.

We did not collect explicit measures of fatigue, nor did we continuously instrument illumination to quantify micro-fluctuations during data collection. While viewing conditions were specified and standardized, these factors may still contribute to individual variability. Accordingly, the present work is framed as an exploratory characterization of perceptual outcomes under controlled setup parameters; future studies can add fatigue assessments, strict time constraints, and continuous monitoring of ambient illumination to further increase stability and reproducibility.

Future work should (1) replicate the present findings on more heterogeneous participant samples (age range, educational background, and cross-cultural/language contexts) to assess generalizability; (2) implement targeted stimulus manipulations designed to probe specific regions of the proposed phenomenal gradient (e.g., graded occlusion to elicit amodal completion; controlled illumination/material cues; and parametrically varied boundary perturbations to modulate event/causal interpretations); (3) refine the protocol by introducing time-constrained reporting, fatigue monitoring, and repeated within-participant trials where appropriate to estimate stability; (4) develop a more controlled AI-side assessment using fixed prompts, repeated runs, and quantitative coding that separates veridical attribute extraction from organization- and event-level interpretations. These steps would convert the present comparative phenomenology into a tightly constrained program of psychophysical and computational tests.

## Data Availability

The raw data supporting the conclusions of this article will be made available by the authors on request.
